# Resveratrol Impairs Glioma Stem Cells Proliferation and Motility by Modulating the Wnt Signaling Pathway

**DOI:** 10.1371/journal.pone.0169854

**Published:** 2017-01-12

**Authors:** Chiara Cilibrasi, Gabriele Riva, Gabriele Romano, Massimiliano Cadamuro, Riccardo Bazzoni, Valentina Butta, Laura Paoletta, Leda Dalprà, Mario Strazzabosco, Marialuisa Lavitrano, Roberto Giovannoni, Angela Bentivegna

**Affiliations:** 1 School of Medicine and Surgery, University of Milano-Bicocca, via Cadore, Monza, Italy; 2 PhD Program in Neuroscience, University of Milano-Bicocca, via Cadore, Monza, Italy; 3 NeuroMI, Milan center of Neuroscience, University of Milano Bicocca, Dept. of Neurology and Neuroscience, San Gerardo Hospital, via Pergolesi, Monza, Italy; 4 PhD Program in Translational and Molecular Medicine (DIMET), University of Milano-Bicocca, via Cadore, Monza, Italy; University of Alabama at Birmingham, UNITED STATES

## Abstract

Glioblastoma multiforme (GBM) is a grade IV astrocytoma and the most common form of malignant brain tumor in adults. GBM remains one of the most fatal and least successfully treated solid tumors: current therapies provide a median survival of 12–15 months after diagnosis, due to the high recurrence rate. Glioma Stem Cells (GSCs) are believed to be the real driving force of tumor initiation, progression and relapse. Therefore, better therapeutic strategies GSCs-targeted are needed. Resveratrol is a polyphenolic phytoalexin found in fruits and vegetables displaying pleiotropic health benefits. Many studies have highlighted its chemo-preventive and chemotherapeutic activities in a wide range of solid tumors. In this work, we analyzed the effects of Resveratrol exposure on cell viability, proliferation and motility in seven GSC lines isolated from GBM patients. For the first time in our knowledge, we investigated Resveratrol impact on Wnt signaling pathway in GSCs, evaluating the expression of seven Wnt signaling pathway-related genes and the protein levels of c-Myc and β-catenin. Finally, we analyzed Twist1 and Snail1 protein levels, two pivotal activators of epithelial-mesenchymal transition (EMT) program.

Results showed that although response to Resveratrol exposure was highly heterogeneous among GSC lines, generally it was able to inhibit cell proliferation, increase cell mortality, and strongly decrease cell motility, modulating the Wnt signaling pathway and the EMT activators. Treatment with Resveratrol may represent a new interesting therapeutic approach, in order to affect GSCs proliferation and motility, even if further investigations are needed to deeply understand the GSCs heterogeneous response.

## Introduction

Glioblastoma multiforme (GBM) is a grade IV astrocytoma and the most common form of malignant brain tumor in adults [1]. Despite improvements in current therapies GBM remains one of the most fatal solid tumors: the median survival is currently 12–15 months after diagnosis, due to the high recurrence rate [2, 3].

One of the factors underlying tumor recurrence and poor long-term survival is the marked intratumoral heterogeneity, mirrored by the presence of distinct sub-populations of cells showing different tumorigenic capabilities [4]. In particular Glioma Stem Cells (GSCs), a small subpopulation of cells with stem-like properties, such as an enhanced self-renewal capacity and a multilineage differentiation potential, are believed to be the real driving force for tumor initiation, progression and relapse [5, 6]. The highly migratory capacity of GSCs is another crucial factor that results in an invasive spread of GBM in different areas of the brain, thus making this tumor extremely difficult to eradicate [1].

Resveratrol (*trans*-3,4’,5 tryhidrostilbene) (RSV) is a natural polyphenolic phytoalexin found in fruits and vegetables, acting as a phytoestrogen and displaying pleiotropic health benefits. This compound has received considerable attention over the last decades, especially for its anti-oxidant and anti-inflammatory properties, which can explain its efficacy in treating cardiovascular diseases and its neuroprotective effect, due to its ability to cross the blood brain barrier [7]. RSV beneficial effects involve the modulation of a huge number of molecular targets, including the activation of enzymes such as sirtuin1 (SIRT1), a NAD-dependent deacetylase, which can influence chromatin remodeling and gene expression [8].

Interest in RSV took a further upsurge when Jang and Pezzuto highlighted its chemo-preventive and antineoplastic activities, demonstrating the efficacy of this compound in all the three major stages of carcinogenesis (initiation, promotion and progression) [9]. Afterwards lots of studies demonstrated its anti-proliferative, pro-apoptotic [10–12] and anti-migratory effects [13–16] in a wide range of human cancer cells. Moreover many epidemiological observations highlighted RSV chemo-preventive properties, indicating that the incidence of some cancers is much lower in people who consume high amounts of this dietary phytoestrogen [17].

Interestingly it has already been observed that RSV can directly or indirectly affect self-renewal pathways, frequently impaired or aberrantly activated through either genetic or epigenetic alterations in cancer stem cells [18]. In particular, RSV was shown to be able to significantly inhibit Wnt signaling pathway, a highly evolutionarily conserved pathway that plays a crucial role in stem cell homeostasis and in different tumors [11, 19, 20], including GBM [21–25].

In this study we investigated the effect of RSV on seven established GSC lines, isolated from GBM patients, evaluating its effect on cell viability, proliferation and motility. For the first time in our knowledge we investigated RSV impact on Wnt signaling pathway in GSCs. We evidenced that RSV differently affects the GSC lines, but, interestingly, in most of the cases it was able to inhibit cell proliferation, increase cell mortality, and strongly decrease cell motility. Moreover, it modulated the expression of Wnt-related genes and induced evident reductions of nuclear β-catenin and c-Myc protein levels. Intriguingly it also caused a decrease of Twist1 and Snail, two main activators of the epithelial-mesenchymal transition (EMT) program.

## Materials and Methods

### Cell lines and cell culture conditions

All the glioma stem cell (GSC) lines used in this work (GBM2, GBM7, G144, G179, G166, GliNS2, GBM04) were isolated from patients affected by glioblastoma and extensively characterized for their stemness properties. GBM2, GBM7, G144, G166, GliNS2, GBM04 derived from classic glioblastoma multiforme, while G179 derived from a giant cell variant glioblastoma. All the GSC lines have been already expanded *in vitro* as stable cell lines and used as powerful model for studying their biology and testing drug susceptibility [26, 27]; furthermore their cytogenomic and epigenomic profiles were well characterized [28].

The stemness properties of these GSC lines were periodically monitored, as already described [29]. Cell expansion was carried out in a proliferation permissive medium composed by DMEM F-12 (Euroclone) and Neurobasal 1:1 (Invitrogen), B-27 supplement without vitamin A (Invitrogen), 2 mM L-glutamine (Euroclone), 10 ng/ml recombinant human bFGF and 20 ng/ml recombinant human EGF (Miltenyi Biotec), 20 UI/ml penicillin and 20 μg/ml streptomycin (Euroclone) (complete medium). GSCs were cultured in adherent culture condition in T-25 cm^3^ flasks coated with 10 μg/ml laminin (Invitrogen), in 5% CO_2_/95% O_2_ atmosphere.

### Drug and treatments

RSV (Sigma, P.M. = 228,24 g/mol) was dissolved in dimethylsulfoxide (DMSO) to make a 100 mM stock solution and then diluted to the final selected concentration (10-50-100-200 μM) with complete cell culture medium. The stock preparation was stored at -20°C. DMSO had no effect on the cell survival. All procedures were carried out in the dark because RSV is photosensitive.

### MTT assay

Cell metabolic activity was assessed by the MTT (3-[4,5dimethylthiazol-2-yl]-2,5-diphenyl tetrazolium bromide) assay in order to evaluate the efficacy of RSV. Cells were seeded in 96 well-plates at a density of 4x10^4^ cells/well in 100 μl of culture medium and incubated at 37°C. After 24 hs, RSV at various concentrations (10-50-100-200 μM) was added to cell culture medium. After the drug incubation time (24, 48 or 72 hs) MTT solution (1 mg/ml, Sigma) was added to each well and cells were incubated for 3 hs at 37°C. Therefore, formazan was solubilized in absolute ethanol and the absorbance of the dye was measured spectrophotometrically with FLUOstar Omega microplate reader (BMG Labtech) at 595 nm. The percentage of inhibition was determined by comparing the absorbance values of drug-treated cells with that of untreated controls: [(treated-cell absorbance/untreated cell absorbance) × 100]. The results reported are the mean values of two different experiments performed at least in triplicate.

### Trypan blue dye exclusion assay

Cells were plated in 60 mm Petri dishes at a density of 1,2x10^6^ cells/dish and cultured overnight. Then, the cells were treated with different concentrations of RSV (10–100 μM) for 48 or 72 hs. Thereafter, the cells were stained using trypan blue dye (Sigma) to count cell numbers and determine the drug cytotoxic/antiproliferative effects. The treated samples were compared with the untreated controls. The results reported are the mean values of two different experiments.

### Mitotic index analysis

The Mitotic Index (MI) was assessed in order to evaluate RSV effect on cell proliferation. 2x10^6^ cells were seeded in T-25 cm^3^ in 5 ml of medium. Subsequently, cells in exponential growth phase were treated with 100 μM RSV for 48 hs. Then metaphase chromosome spreads were obtained using standard procedures as previously described [28].

The chromosomes were QFQ-banded using quinacrine mustard (Roche) and slides were mounted in McIlvaine buffer. Slides were analyzed using Nikon Eclipse 80i fluorescence microscope (Nikon) equipped with a COHU High Performance CCD camera. MI was evaluated counting the percentage of mitosis scoring at last 1000 nuclei. Data were obtained as mean values derived from two independent experiments.

### Wound healing assay

To evaluate cell motility, cells were plated in 6-well plates with laminin coating in proliferative permissive medium and grown to confluence. Cells were growth-arrested for 24 hs in a medium without growth factors. Then a sterile tip was used to create a scratch in the cell layer and images were captured (0 hs time point). Therefore cells were treated with various concentrations of RSV (10-50-100-200 μM) and pictures were taken after 48, 72 and 96 hs to evaluate wound closure. This test was not performed on the G166 cell line because, despite the long time of cultivation, cells did not grow to confluence.

Since RSV is photosensitive different fields were recorded for each time point. Matching untreated control cultures were also assessed. Wounds were evaluated using TScratch freeware software (http://www.cse-lab.ethz.ch/), which calculated the fraction of open image area at a later time point compared to the initial time point. The migration distances were expressed as percentages over control values and were calculated as wound area at a given time compared to the initial wound surface.

### Invasion assay

The cell invasion assay was performed using a Boyden chamber with a gelatin-coated polycarbonate filters with 8 μm pore size (NeuroProbe). Briefly 5x10^3^ cells, untreated or treated with RSV 100 μM for 96 hs, were seeded in the upper compartment of the chamber with serum-free medium. Medium with 10% or 20% (for G179 cell line) FBS was added into the lower compartment. After 24 hs of culture at 37°C cells that did not migrate were removed from the upper face of the filters, while cells on the lower surface of the membrane were fixed in methanol and stained with eosin G and tetrazolium blue chloride. Photographs were taken and the number of migrated cells was quantified using Image J software. The experiments were performed in triplicate.

### RNA extraction

RNA extraction from untreated and 100 μM RSV 96 hs treated cells was performed using the miRNeasy Mini Kit (Qiagen), according to the manufacturer’s protocol.

### Real time PCR array

RT^2^Profiler PCR Arrays (Qiagen) was assessed on untreated and 100 μM RSV 96 hs treated cells in order to evaluate RSV effect on the expression of genes involved in Wnt pathway. RNA samples from treated and untreated cells were converted into first-strand cDNA using the RT^2^ First Strand Kit (Qiagen). Then, RT^2^ Profiler PCR Arrays were assessed according to the manufacturer’s protocol using a 12 wells array PCR custom, containing primers for 7 Wnt pathway-focused genes (*WNT1*, *FZD4*, *CTNNB1*, *EP300*, *CREBBP*, *TCF7*, *MYC*), and for 2 housekeeping genes (*HPRT1*, *TBP*). Briefly the cDNA was mixed with an appropriate RT SYBR Green Mastermix. This mixture was aliquoted into the wells of the PCR Array and PCR was performed by means of a real time cycler (Applied Biosystems) properly programmed (1 cycle: 10 min 95°C, 40 cycles: 15 s 95°C, 1 min 60°C). Relative gene expression was determined using data from the real-time cycler and the ΔΔCt method. The cut-off values for gene expression fold changes was established at ± 1,5: values ≥ +1,5 indicate gene upregulation, while values ≤ -1,5 indicate gene downregulation. The gene expression fold changes data were obtained as mean values derived from two independent experiments.

### Protein extracts and western blotting

For Myc proto-oncogene protein evaluation, cells were treated for 72 hs with RSV 100 μM. At the end of the treatment cells were trypsinized, collected and lysed using the following buffer: TrisHCl 50 mM, NaCl 500 mM, EDTA 1 mM, EGTA 1 mM, NP-40 0,5% (vol/vol), Protease inhibitor cocktail (Sigma Aldrich) 2% (vol/vol), Phosphatase Inhibitor Cocktail #2 (Sigma Aldrich) 1% (vol/vol), Phosphatase Inhibitor Cocktail #3 (Sigma Aldrich) 1% (vol/vol). Lysates were quantified with Micro-BCA assay (Thermo Scientific), following manufacturer’s instructions; 15 μg of protein lysates were loaded on NuPAGE Bis-Tris pre-cast mini gels (Life Technologies). At the end of the run, gels were then blotted on Nitrocellulose membranes using iBlot 2 (Life Technologies). After blocking, membranes were incubated with the following primary antibodies: anti c-Myc (Rabbit Monoclonal, Cell Signaling Technology [D84C12]), anti-GAPDH (Mouse Monoclonal, Sigma-Aldrich [GAPDH-71.1]. Secondary HRP-conjugated antibodies used were: ECL™Anti Rabbit and ECL™Anti Mouse (GE Healthcare). Western Blots images were digitally acquired with G-BOX (Syngene). Quantitative densitometry of Western Blot was performed with ImageJ 1.49q (Rasband, W.S, U. S. National Institutes of Health, Bethesda, Maryland, USA, http://imagej.nih.gov/ij/, 1997–2014). The results are expressed as means of four independent experiments. Two-tailed paired t-test was used to detect significant differences in protein expression between Control and Treated groups (GraphPad Prism 6, GraphPad Software, Inc.); *p*-value<0,05 was considered as significant.

For Catenin beta-1 (or β-catenin), Twist-related protein 1 (or Twist1) and Zinc finger protein SNAI1 (or Snail1), cells were treated for 96 hs with RSV 100 μM. At the end of the treatment cell lysate from nuclear and cytoplasmic fractions (NePer, Pierce) were obtained as already described [30]. Briefly, equal concentrations of protein lysate obtained from cultured cells, previously evaluated by Bradford assay (Sigma-Aldrich), were electrophoresed on a 4–12% NuPAGE Novex Bis-Tris gel (Life Technologies) with MES (NuPage Novex, Life Technologies) and proteins were transferred to a nitrocellulose membrane (Life Technologies). The membrane was then blocked with 5% non-fat dry milk (Euroclone) in Tris-buffered saline (TBS) supplemented with 0.1% Tween-20 (TBS-T) for 1 hour and then incubated overnight at 4°C with mouse anti-β-catenin (1:1000, BD biosciences), goat anti-Snail1 (1:1000, AbCam), and rabbit anti-Twist1 (1:500, Santa Cruz Biotechnology), diluted in TBS-T + 5% non-fat dry milk. As reference protein, rabbit anti-Histone 3 (1:2000, Sigma-Aldrich) or mouse anti-GAPDH (1:10000, Santa Cruz Biotechnologies) were used. The membrane was washed three times with TBS-T before incubation with anti-rabbit (1:2000, Bio-Rad), anti-mouse (1:2000, Sigma-Aldrich), or anti-goat (1:5000, Santa Cruz Biotechnology) HRP-conjugated secondary antibodies for 1 hour. Proteins were visualized using SuperSignal West Pico or Dura chemiluminescent substrate (Thermo Scientific) with a Kodak Image Station 440 CF (Eastman Kodak Co., New Haven, CT, USA). Bands were then quantified with ImageJ (https://imagej.nih.gov/ij) and results were normalized versus controls.

### Statistical analysis

Statistical analysis was carried out performing Yates’ chi-square test (Mitotic Index) or t-test (MTT assay, Trypan blue assay, Wound healing assay, Boyden chamber assay, Western blot) on raw data, by means of Excel spreadsheet (Microsoft Office 2013, Microsoft Corporation). The critical level of significance was set at *p<*0,05.

## Results

### Sensitivity and resistance to RSV measured by metabolic activity

**Fig 1:**
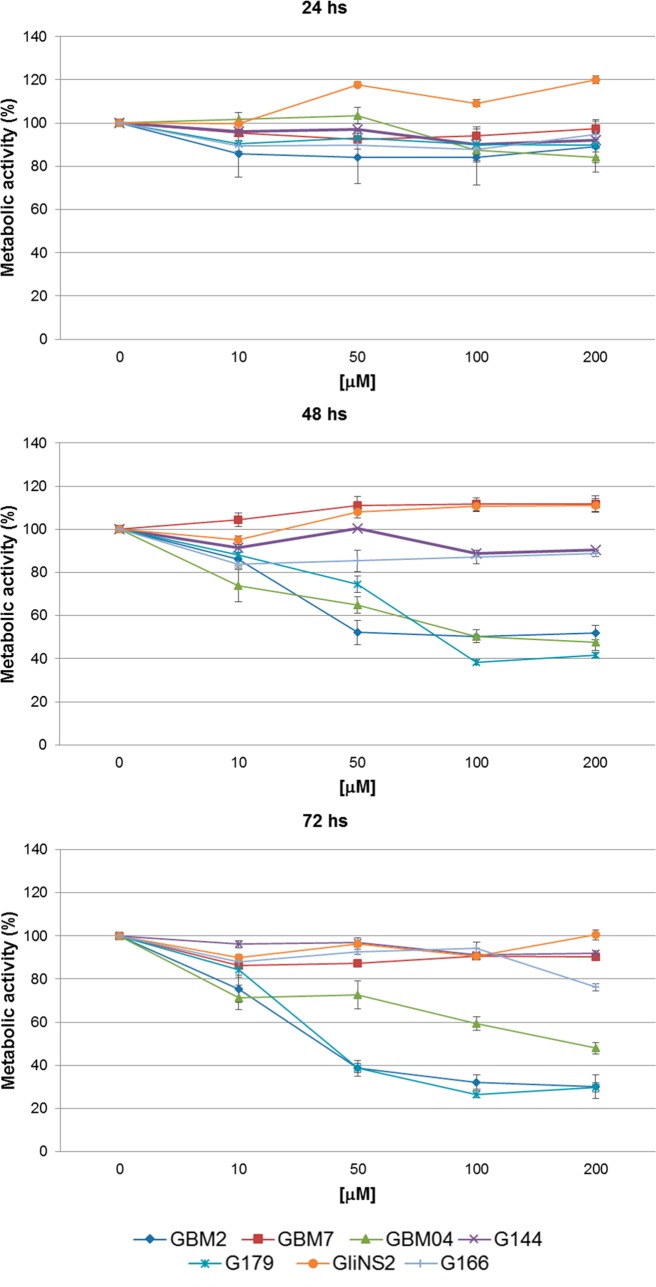
RSV effect on GSC metabolic activity. Metabolic activity was analyzed by MTT assay. Results represent the means from two different experiments performed at least in triplicate and are reported as percentage of drug-treated cells relative to untreated cells. Bars represent SEM.

The effect of RSV on the metabolic activity was determined by means of MTT assay (Fig 1, S1 Table). Metabolic activity values were almost unchanged in all GSC lines after 24 hs of treatment; on the contrary after 48 hs, GBM2, G179 and GBM04 cell lines showed a significant decrease in metabolic activity (*p<*0,05), at the highest doses of RSV in a dose-dependent manner, compared to the matching untreated cells. Otherwise G166 and G144 showed a very faint decrease, and GBM7 and GliNS2 showed even an increase. Exposure to increasing doses of RSV for 72 hs resulted again in a progressive reduction of the metabolic activity in GBM2 and G179 cell lines, while GBM04 did not appear to be affected by the prolongation of the treatment. Metabolic activity values of GBM7 and GliNS2 remained stable after 48 hs and 72 hs of treatment. G144, GBM7, GliNS2 and G166 could, therefore, be considered resistant to the treatment.

### RSV effect on GSC viability

**Fig 2:**
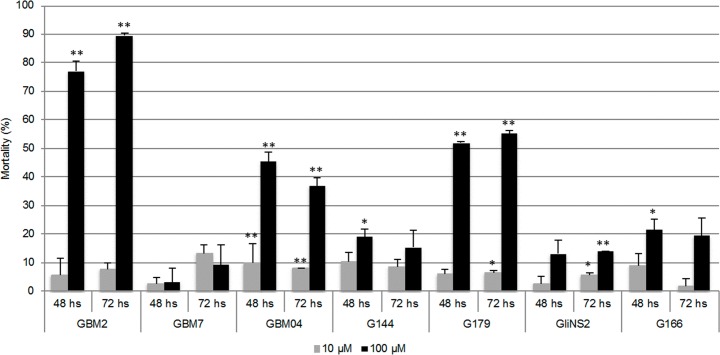
RSV effect on GSC viability. Cell viability was analyzed by Trypan blue dye exclusion assay after exposure to two different concentrations of RSV (10–100 μM) for 48 and 72 hs. Results are reported as percentage of cell mortality in drug-treated cells relative to untreated cells and are the means of two different experiments ±SEM. T-test on raw data: * *p<*0,05; **p<0,001.

GSCs viability was evaluated by means of the Trypan blue dye exclusion assay. The administration of 10 μM RSV didn’t induce any relevant changes in GSCs viability after both 48 and 72 hs. Again, GBM2, G179 and GBM04 were the most sensitive cell lines after 48 hs of treatment with 100 μM RSV, showing a significant increase in the percentage of cell mortality (Fig 2). 72 hs of exposure resulted in a progressive increase in cell death in GBM2 cell line, while G179 and GBM04 cell line seemed not to be affected by the prolongation of the treatment. All the other cell lines showed lower cell mortality after both 48 hs and 72 hs of 100 μM RSV treatment. These data suggested that in responsive GSCs, RSV had mainly a cytotoxic effect, which was definitely more evident at the highest drug concentration.

### RSV effect on GSC proliferation

**Fig 3:**
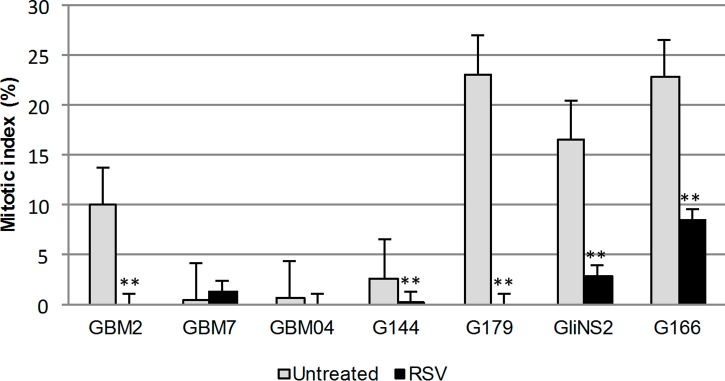
RSV effect on GSC proliferation. Cell proliferation was evaluated through the determination of the mitotic index. Results are reported as percentages from the means of two independent experiments ±SEM. Yates’ Chi-square test on raw data: * *p<*0,01; ** *p<*0,001.

In order to study the effect of RSV exposure on cell proliferation, we evaluated the Mitotic Index (MI). 100 μM RSV for 48 hs revealed a significant decrease of MI in five out of seven cell lines (Fig 3). In particular, in GBM2 and G179 cell lines, which had already been shown to be very sensitive to the drug treatment, the MI was almost zero after RSV administration. GBM04 and GBM7 cell lines didn’t show any statistically significant change in MI, probably because the proliferation rate was extremely low even in the untreated cells.

### RSV inhibited GSC motility and invasion

**Fig 4:**
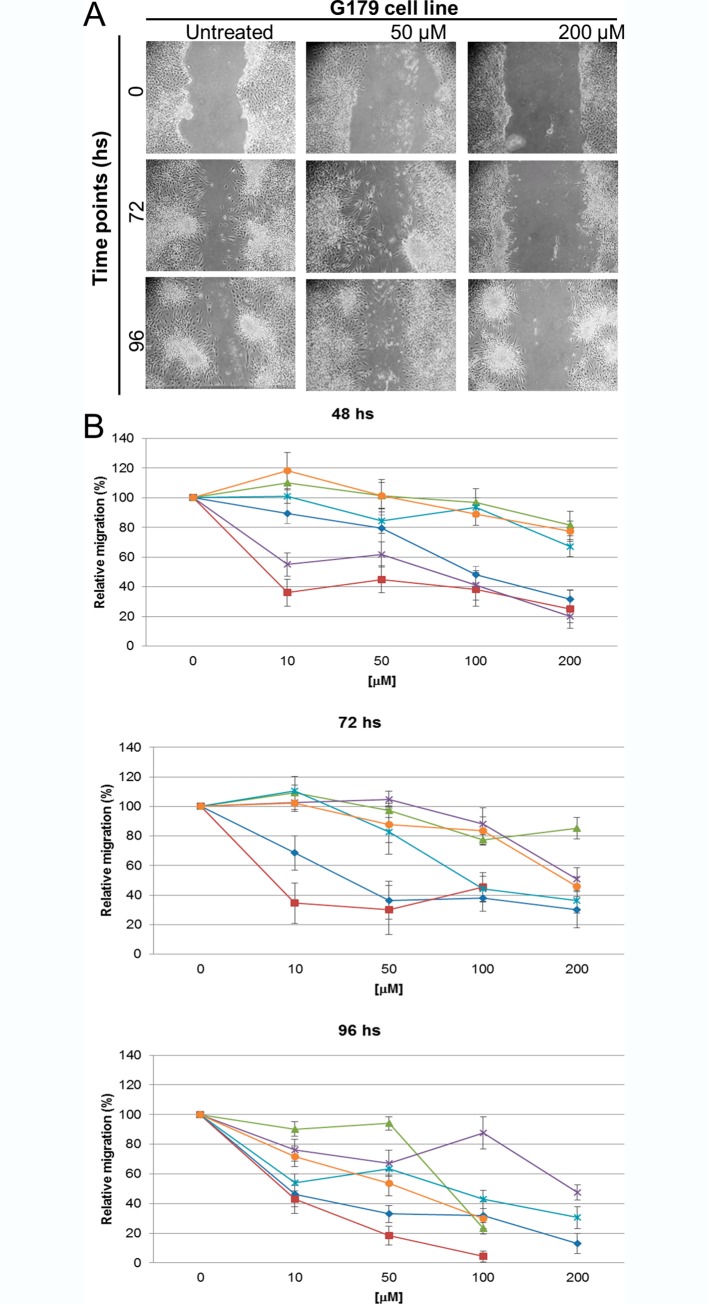
RSV reduced GSC motility. The migration ability of GSCs was investigated by Wound healing assay. Monolayers of growth-arrested GSCs were scraped and treated with different concentrations of RSV for 48, 72 and 96 hs. (A) Representative images were taken at different time points to evaluate wound closure. (B) The migration was quantified by means of the TScratch software and results are reported as percentages over control values and calculated as the wound area at a given time compared to the initial wound surface. Bars represent SEM.

The effect of RSV on cell motility was firstly investigated using the wound healing assay. The RSV treatment strongly reduced cellular motility in all GSC lines (Fig 4), in a dose- and time- dependent manner, compared to the untreated cells (S2 Table). A significant inhibition of motility started as early as after 48 hs of treatment for all GSC lines. G144, GBM7 and GBM2 cell lines showed a significant reduction especially at the highest doses of RSV treatment (100–200 μM) (*p<*0,01). The inhibition of GSCs migration was even more evident after 72 and 96 hs. An interesting behavior was registered for GBM04. In this cell line, the migration slightly decreased only at the higher dosages after 48 and 72 hs, while after 96 hs of RSV exposure cells showed a significant reduction of motility (*p<*0,0001). However, the treatment with the highest dose of RSV for 96 hs, in GBM04, GliNS2 and GBM7 cell lines did not allow us to obtain an adequate number of cells to conduct the experiment, probably because of the prevailing cytotoxic effect of the drug and the extremely low rate of proliferation of these cell lines. GBM7 showed this behavior even after 72 hs of exposure to the highest drug concentration.

**Fig 5:**
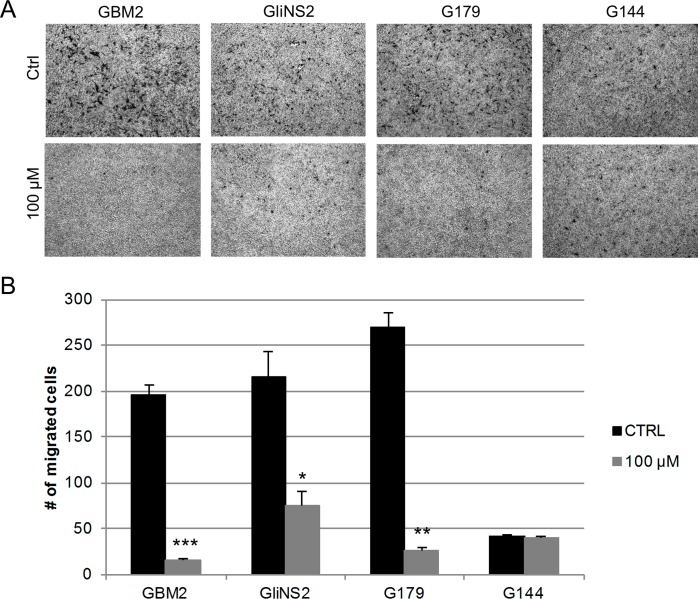
RSV effect on GSC invasive behavior. Invasion of GSCs was determined using gelatin-coated Boyden chambers on 4 GSC lines. GSCs, untreated or treated with RSV 100 μM for 96 hs, were seeded in the upper compartment of the chamber and induced to migrate using FBS as chemoattractant in the lower compartment. After 24 hs of incubation photographs were captured and the number of migrated cells was quantified using Image J software. Results are reported as the mean number of invaded cells from three different experiments. Bars represent SEM. * *p<*0,05; ** *p<*0,01; ***p<0,001.

The RSV effect on cell migration was confirmed evaluating GSC invasive ability after treatment with RSV 100 μM for 96 hs by means of a Boyden chamber assay. This assay was performed on two RSV-sensitive (GBM2, G179) and on two RSV-resistant (GliNS2, G144) GSC lines, on the basis of the response in the metabolic and in the migration assays. GBM2, G179 and GliNS2 cell lines showed a dramatic reduction of the number of migrated cells (*p<*0,05) (Fig 5). On the contrary in G144 RSV exposure did not induce a relevant decrease of the motility, supporting the data obtained from the wound healing assay.

### RSV effect on Wnt signaling pathway

In order to unravel the molecular mechanism leading to the GSCs response, with particular regards to migration ability behavior, to RSV treatment, the WNT signaling expression profile was performed using Wnt-specific PCR arrays on untreated and 100 μM RSV treated cells for 96 hs.

**Fig 6:**
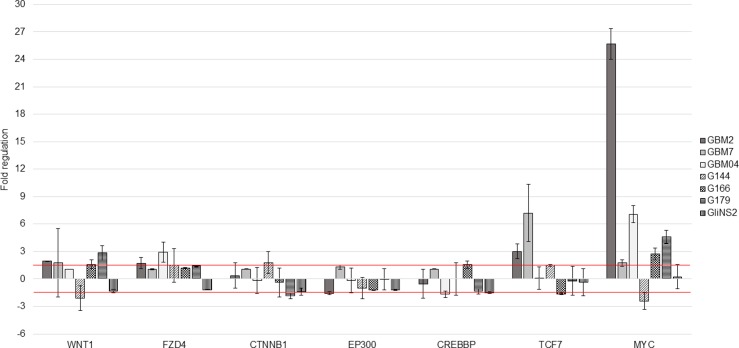
Fold regulation of 7 Wnt signaling pathway-related genes. Expression variations were evaluated after 96 hs of exposure to RSV 100 μM in 7 GSC lines. Bars represent SEM. Red lines represent the cut-off range (±1,5): fold regulation values ≥ +1,5 indicate gene upregulation, while values ≤ -1,5 indicate gene downregulation.

Results showed that RSV was able to modulate Wnt signaling pathway in all GSC lines (Fig 6). In particular, the expression of the ligand *WNT1* and the downstream oncogene *MYC* simultaneously changed in five out of seven GSC lines and often in the same direction. In GBM2, GBM7, G166, G179 cell lines both *WNT1* and *MYC* were upregulated, while in G144 cell line these two genes were downregulated by RSV treatment. The expression levels of *FZD4*, *TCF7* and *CREBBP* changed only in at least three out of seven GSC lines after RSV exposure. *CTNNB1*, the most important effector of Wnt signaling pathway, and *EP300*, generally showed unchanged expression levels.

**Fig 7:**
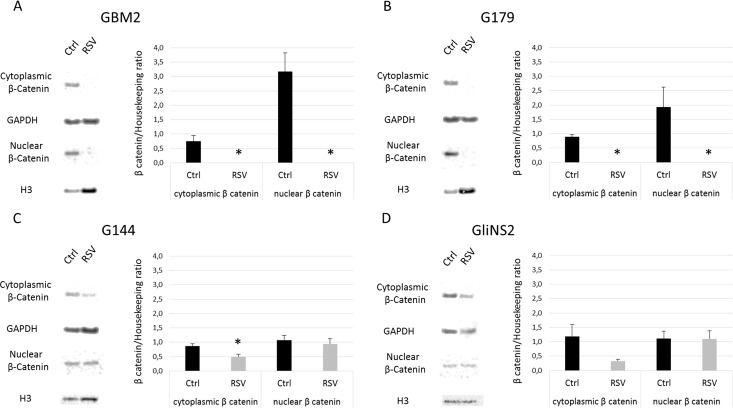
Cytoplasmic and nuclear β catenin protein levels after 100 μM RSV administration for 96 hs. Representative images of Western blot analysis of cytoplasmic and nuclear β-catenin in GBM2 (A), G179 (B), G144 (C) and GliNS2 (D) cells and quantitative densitometry analysis of at least three independent experiments. GAPDH and H3 Histone were used as loading controls. Cytoplasmic and nuclear β-catenin protein levels were normalized on GAPDH and H3 Histone, respectively, and then expressed in Arbitrary Units (AU). Bars represent SEM. * *p<*0,05.

**Fig 8:**
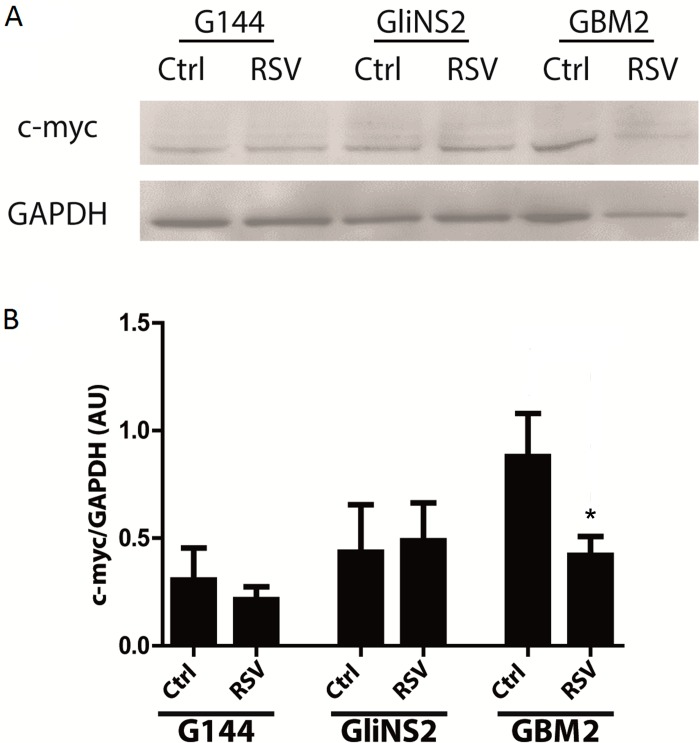
c-Myc protein levels were downregulated by RSV administration in GBM2 cells. (A) Representative images of Western blot analysis of c-Myc on G144, GliNS2 and GBM2 cells total protein extracts. GAPDH was used as loading control. (B) Quantitative densitometry analysis of 4 independent experiments. c-Myc protein levels were normalized on GAPDH and expressed in Arbitrary Units (AU). Bars represent SEM. Indicated p-values were obtained after two-tailed paired t-test. * *p<*0,05.

Since the protein levels and localization of Catenin Beta-1 (or β catenin), which is encoded by *CTNNB1* gene, are crucial indicators of WNT pathway activity, we analyzed them by Western blot, investigating the cytoplasmic and nuclear protein fractions of two RSV-sensitive (GBM2 and G179) and two RSV-resistant cell lines (GliNS2 and G144). With regard to the cytoplasmic protein levels, after 96 hs of 100 μM RSV treatment, we observed a general downregulation in all the cell lines analyzed. In particular, in GBM2 and G179 we obtained a complete depletion of the protein (*p<*0,01) (Fig 7A and 7B), while G144 and GliNS2 showed consistent reductions of 42,1% (p<0,05) and 70,1%, respectively (Fig 7C and 7D). Afterwards, we evaluated the nuclear levels of the Catenin Beta-1, which is the functional and active form: in GBM2 and G179 we had once again a complete depletion (Fig 7A and 7B), but the other two cell lines showed no differences between untreated and RSV-treated samples (Fig 7C and 7D). Then, to investigate if the observed transcriptional upregulation of *MYC* induced by RSV was associated to an increase in the c-Myc protein expression, protein extracts of 3 GSC lines (G144, GliNS2 and GBM2), which highlighted different alterations in the *MYC* gene expression levels, were analyzed by western blot. As shown in Fig 8A and 8B, RSV administration caused a significant reduction of c-Myc protein in GBM2 cell lines as compared to untreated cells (*p =* 0,0197). Contrarily, no significant effect was observed in G144 or in GliNS2 cells.

### RSV downregulated epithelial mesenchymal transition markers

**Fig 9:**
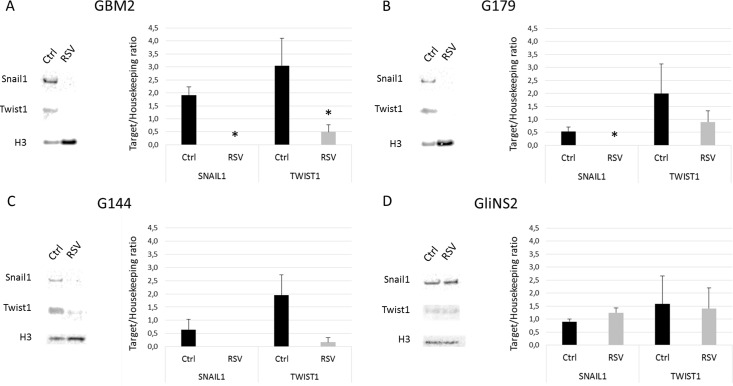
Twist1 and Snail1 protein levels after 100 μM RSV administration for 96 hs. Representative images of Western blot analysis of nuclear Twist1 and nuclear Snail1 in GBM2 (A), G179 (B), G144 (C) and GliNS2 (D) cells and quantitative densitometry analysis of at least three independent experiments. H3 Histone was used as loading controls. Nuclear Twist1 and nuclear Snail1 protein levels were normalized on H3 Histone and then expressed in Arbitrary Units (AU). Bars represent SEM. * *p<*0,05.

Since it is known the role of Wnt pathway in glioblastoma stem cells maintenance, migration and invasion as well as in drug resistance [31] and given our results on the modulation of Wnt pathway in RSV-treated GSCs, we wanted to investigate the effect of RSV treatment on epithelial-mesenchymal transition (EMT) program. To this extent, we analyzed Snail1 and Twist1 protein levels by western blot on the nuclear protein extracts of four GSC lines (GBM2, G179, G144 and GliNS2) treated with 100 μM RSV for 96 hs compared to untreated samples. In three out of four cell lines RSV strongly downregulated the expression of the proteins tested, while GliNS2 cell line didn’t show any consistent variation after treatment (Fig 9).

## Discussion

Glioblastoma multiforme (GBM) is the most common and malignant tumor of the central nervous system [2, 3]. It is characterized by the presence of different subpopulation of cells with different grade of differentiation, including a Glioma Stem Cells (GSCs) population characterized by an enhanced self-renewal, an elevated invasive behavior and the ability to drive tumor formation, maintenance and progression [6]. Given the failure of existing treatments, multiple efforts to search for new therapeutic strategies for the eradication of the stem cell subpopulation are ongoing in order to achieve an effective treatment for this tumor.

In this study we analyzed for the first time the efficacy of RSV on 7 established GSC lines from GBM, whose cytogenetic, genomic and epigenomic profiles were extensively characterized [28]. GSC lines have been already demonstrated to be a valuable *in vitro* model for widening the understanding on the biology of GBM, as they mirror the characteristics of the primary tumor, and testing drug susceptibility [25, 28, 32].

RSV is a polyphenolic compound synthesized by a lot of plant in response to injury, UV irradiation and fungal attack [33]. Accumulating evidences indicated that RSV is able to inhibit multiple cellular events associated with tumor initiation, promotion, and progression, and, therefore, might be a promising chemopreventive and chemotherapeutic agent [9, 34]. Accordingly, the U87 and U251 glioma cell lines have been reported to respond to this drug with cell cycle arrest, induction of autophagy and apoptosis [34, 35] and RSV has been shown to suppress the angiogenesis and tumor growth of gliomas even *in vivo* [36, 37]. Moreover this drug might be useful for the treatment of brain tumors because of its ability to cross the blood brain barrier [38].

In order to characterize the antineoplastic effects of RSV in our GSC lines we evaluated, first of all, cell metabolic activity, viability and proliferation after drug administration.

MTT assays revealed a significant reduction of cell metabolic activity in three cell lines out of seven (GBM2, G179 and GBM04) especially after the longer times of regimens. These data were also confirmed by Trypan blue dye exclusion assays which suggested that in these three cell lines RSV had mainly a cytotoxic effect, which was generally dose-dependent and, only in GBM2, also time-dependent.

RSV effect on cell proliferation was evaluated through MI determination. This parameter has very important clinical implications because the mitotic activity is a crucial property related to the tumor aggressiveness. This parameter showed a marked reduction in five cell lines out of seven. In particular, GBM2 and G179 cell lines, which already highlighted a significant inhibition of metabolic activity and viability, revealed also a drastic decrease of the MI. Contrariwise GBM7 and GBM04 cell lines did not show any significant change in MI, probably because of the extremely low proliferation rate, even in the untreated cells.

Since one of the hallmarks of GSCs is the elevated infiltrative behavior, we analyzed their motility by means of a migration and an invasion assay after RSV treatment. We demonstrated that the motility of all the examined GSC lines was greatly impaired by RSV. Interestingly this inhibition was highlighted in the Wound healing assay as early as after 48 hs of drug exposure and confirmed by the invasion assay, where the inhibitory effect of exposure to 100 μM RSV for 96 hs was highly significant in GBM2, G179 and GliNS2 cell lines. Taken together all these findings are in accordance with a huge number of studies on RSV inhibitory effect on glioblastoma cell migration and invasion, but for the first time we confirmed it in a comprehensive panel of GSC cell lines [16, 39, 40].

Another feature of GSCs is the aberrant activation of several embryonic signaling pathways, such as Wnt signaling pathway, which has been reported to regulate self-renewal of GSCs, as well as migration and differentiation [41–43]. Therefore, for the first time in our knowledge, we evaluated RSV ability to modulate Wnt signaling pathway in GSCs, analyzing the expression of 7 genes (*WNT1*, *FZD4*, *CTNNB1*, *EP300*, *CREBBP*, *TCF7*, *MYC*).

Previous studies demonstrated that TCF7 transcriptional factor, and p300 and CREBBP coactivators, play a key role in controlling the switch between self-renewal and differentiation [44, 45]. In particular it has been shown that in GBM cells the transcriptional co-activator p300 regulates cell differentiation, activating GFAP and repressing Nestin genes [46]. RSV did not induce any significant change in *TCF7*, *EP300* and *CREBBP* expression levels in our GSCs.

With regard to *CTNNB1* expression levels, five out of seven GSC lines did not show any relevant variations, while G179 and G144 displayed opposite modulation trends. As β catenin, the product of *CTNNB1* gene, is the most important effector of the Wnt signaling pathway, we decided to evaluate also its protein levels on cytoplasmic and nuclear extracts. We selected two RSV-sensitive (GBM2 and G179) and two RSV-insensitive (G144 and GliNS2) cell lines in order to relate their behavior with β catenin protein levels. Intriguingly, nuclear β catenin was completely depleted in GBM2 and G179, but it did not vary in the RSV-insensitive cell lines. Thus, we can conclude that the *CNTNNB1* expression levels after RSV treatment were not correlated to the nuclear protein levels of β catenin. Furthermore, we want to highlight that the RSV-sensitive cell lines showed a higher nuclear β-catenin/Histone H3 optical density ratio than RSV-insensitive ones. This suggests that RSV effects are mediated by its inhibitory action on Wnt canonical pathway. Otherwise, low level of nuclear β-catenin could correlate to resistance to RSV.

Surprisingly the Wnt signaling pathway target gene *MYC* showed an increased transcriptional activity in five out of seven cell lines, especially in GBM2; moreover, four cell lines with this alteration showed also the same variation in the expression of the upstream gene *WNT1*. However, RSV drastically decreased c-Myc protein level in GBM2 cell line and these findings are consistent with the RSV induced anti-proliferative effect and suggest that the transcriptional up-regulation of *MYC* gene could be due to a feedback mechanism in cells lacking of c-Myc protein. On the other hand, the relevant decrease in c-Myc protein level could be due to the activation of post-translational regulatory mechanisms. In particular it has been demonstrated that Sirt1, which is strongly activated by RSV, deacetylates and affects c-Myc stability, with a negative feedback loop [47].

Epithelial-mesenchymal transition (EMT) is one of the processes involved in GBM dissemination and chemo-resistance [48, 49]. Interestingly, several studies highlighted that the same molecular players that play a key role in EMT of epithelial tumors are also involved in the regulation of motility of non-epithelial tumors, such as GBM [50, 51]. Hence, we analyzed the nuclear protein levels of Twist1 and Snail1, two transcription factors important for mesodermal differentiation in embryos and for the induction of EMT in cancers [52]. We observed a strong decrease after RSV exposure of the nuclear levels of Twist1 and Snail1 in three out of the four cell lines analyzed. These data suggest that RSV was able to inhibit the activation of EMT program, through the downregulation of Twist1 and Snail1. All these findings strongly support the link between EMT and Wnt signaling pathway in GBM, as after treatment nuclear β-catenin and EMT activators protein levels correlated each other. These data remind previously published data by Kahlert et al., who showed that the modulation of Wnt signaling altered the expression of EMT activators suggesting a crucial role of this process in the regulation of glioma motility [53].

Collectively our results showed, first of all, a wide variability in biological response towards the treatment with RSV across our GSC lines suggesting that this drug might be effective on the proliferation and viability only for a subset of GSC lines. This variability could be partly due to the different genetic background of these cell lines and thus represents a reflection of one of the hallmarks of GBM that is the extreme inter-tumor heterogeneity. Determine the reasons of this heterogeneity may be useful in a future context of a personalized treatment of GBM with RSV.

In order to verify if a different genomic background could explain the variation across our GSCs, we firstly evaluate the distinct signature of copy number alterations comparing the data from a previously performed aCGH analysis for GBM2, G179, G166, GliNS2 and GBM7 cell lines [29]. Intriguingly we observed that GBM2 and G179 cell lines, which showed a significant decrease in the metabolic activity and viability, shared some copy number alterations that are not found in the RSV-insensitive GSC lines, such as the evident mosaic losses of 13q12.11-q34 or 19q13.12–1.43 [28]. It would be interesting to evaluate if in these altered genomic regions there are genes whose aberrant dosage might influence RSV-sensitivity.

RSV had a significant anti-proliferative and anti-migratory effect in GSCs, usually in a dose- and time-dependent manner. It was able to modulate the expression of Wnt signaling pathway-related genes, decreasing nuclear β catenin levels and inducing a transcriptional upregulation of *MYC*, but interestingly it was also able to induce a drastic decrease of c-Myc protein level. Finally, RSV downregulated nuclear Twist1 and Snail1, suggesting its role as a novel anti-EMT compound.

Treatment with RSV may represent a new interesting therapeutic approach, especially for its ability to reduce GSC motility and invasion, eventually impairing the diffuse infiltration of tumor cells in brain tissue, which is one of the main causes of GBM treatment failure. However, investigations are certainly needed in order to deeply understand the GSC heterogeneous response.

## Supporting Information

S1 Table Statistical analysis (*p*-values, t-test) of the effects of RSV on cell viability. *p*-values are referred to the specific treatment compared to the respective untreated cells.
(DOCX)

S2 Table Statistical analysis (*p*-values, t-test) of the effects of RSV on cell motility. *p*-values are referred to the specific treatment compared to the respective untreated cells.
(DOCX)
