# Supplementary material for: Resveratrol Impairs Glioma Stem Cells Proliferation and Motility by Modulating the Wnt Signaling Pathway
Source: PLoS One. 2017 Jan 12;12(1):e0169854. doi: 10.1371/journal.pone.0169854 (PMC5231344; doi:10.1371/journal.pone.0169854)
Supplement: S2 Table — p-values are referred to the specific treatment compared to the respective untreated cells. (DOCX) [file pone.0169854.s002.docx]

| RSV treatment | | | | | | | | | | | | |
| --- | --- | --- | --- | --- | --- | --- | --- | --- | --- | --- | --- | --- |
| Dose | **10 μM** | | | **50 μM** | | | **100 μM** | | | **200 μM** | | |
| Time (hs) | **48** | **72** | **96** | **48** | **72** | **96** | **48** | **72** | **96** | **48** | **72** | **96** |
| GBM2 | n.s. | n.s. | .01 | n.s. | .05 | .001 | .0001 | .01 | .001 | .0001 | .01 | .0001 |
| GBM7 | .01 | .05 | .001 | .05 | .05 | .0001 | .01 | .05 | .0001 | .01 | n.p. | n.p. |
| GBM04 | n.s. | n.s. | n.s. | n.s. | n.s. | n.s. | n.s. | .05 | .0001 | n.s. | n.s. | n.p. |
| G144 | .001 | n.s. | n.s. | .01 | n.s. | .05 | .001 | n.s. | n.s. | .0001 | .01 | .01 |
| G179 | n.s. | n.s. | .001 | n.s. | n.s. | .01 | n.s. | .001 | .0001 | .01 | .0001 | .0001 |
| GliNS2 | n.s. | n.s. | .05 | n.s. | n.s. | .001 | n.s. | n.s. | .0001 | .05 | .0001 | n.p. |

**Table S2. Statistical analysis (*p*-values, t-test) of the effects of RSV on cell motility**. *p*-values are referred to the specific treatment compared to the respective untreated cells. n.s.= not statistically significant; n.p.= not performed
